# Signal Transduction Pathways of EMT Induced by TGF-β, SHH, and WNT and Their Crosstalks

**DOI:** 10.3390/jcm5040041

**Published:** 2016-03-28

**Authors:** Jingyu Zhang, Xiao-Jun Tian, Jianhua Xing

**Affiliations:** Department of Computational and Systems Biology, School of Medicine, University of Pittsburgh, Pittsburgh, PA 15260, USA; zhangjy7@pitt.edu (J.Z.); xjtian@pitt.edu (X.-J.T.)

**Keywords:** signaling transduction pathways, EMT, TGF-β, SHH, WNT, modeling

## Abstract

Epithelial-to-mesenchymal transition (EMT) is a key step in development, wound healing, and cancer development. It involves cooperation of signaling pathways, such as transformation growth factor-β (TGF-β), Sonic Hedgehog (SHH), and WNT pathways. These signaling pathways crosstalk to each other and converge to key transcription factors (e.g., SNAIL1) to initialize and maintain the process of EMT. The functional roles of multi-signaling pathway crosstalks in EMT are sophisticated and, thus, remain to be explored. In this review, we focused on three major signal transduction pathways that promote or regulate EMT in carcinoma. We discussed the network structures, and provided a brief overview of the current therapy strategies and drug development targeted to these three signal transduction pathways. Finally, we highlighted systems biology approaches that can accelerate the process of deconstructing complex networks and drug discovery.

## 1. Biomedical Significance of EMT

Epithelial cells are differentiated cells characterized by uniform cell shape, apical-basal polarity, strong cell-cell adherent junctions, and cell-matrix hemidesmosomes, and limited mobility. Based on these characteristics, epithelial cells normally form single-layered tubes or sheets to cover body, organs, and compose glands [1,2]. On the contrary, mesenchymal cells have front-back polarity and loose cell attachment. They typically have much higher mobility, which is closely related to their regeneration function [3,4]. Mesenchymal cells reside in lymphatic, circulatory, and some connective tissues, and give rise to some other types of cells in these tissues [5,6].

In 1968, Hay noticed that during chicken embryonic development, epithelial cells undergo differentiation and dedifferentiation several times, as well as migrate a relatively long distance within the body. All of these processes require inter-conversion between epithelial and mesenchymal cell phenotypes, called epithelial-to-mesenchymal transition (EMT), and its reverse process, mesenchymal-to-epithelial transition (MET) [7]. Subsequent studies showed that EMT and MET are fundamental in amniotes’ gastrulation and neural crest formation, and generation of body patterns. Specifically, during mammalian embryonic development, several rounds of EMT companied by MET take place, which are generally assigned as primary, secondary, and tertiary EMT based on the developmental stages [8]. The primary EMT occurs in early embryonic development, such as parietal endoderm formation, mesoderm formation, and neural crest delamination. Signaling molecules that initialize and regulate the primary EMT include the transforming growth factor-β (TGF-β) superfamily (e.g., BMP, Nodal, *etc.*), the WNT family [9,10], and the fibroblast growth factor (FGF) family [11]. Two members in the SNAIL family, SNAIL1 and SNAIL2, are important during human early embryonic development for repressing E-cadherin and weakening cell-cell junctions [12]. Mesodermal cells generated from the primary EMT undergo MET subsequently and form secondary epithelial structures, such as the notochord and the somites. The transient epithelial structures go through the secondary EMT and give rise to more differentiated structures, such as endocardial progenitors and connective tissues. Some of the epithelial or mesenchymal structures from the secondary EMT-MET process complete their final differentiation and maintain their phenotypes, provided there is no additional inducing signals. Others continue on a tertiary EMT (and MET), again controlled by multiple signaling pathways, and eventually give rise to complex organs, such as the lung [13] and heart [14].

In wound healing, the initial input signals are from injury. Transcription factors, such as ERK, SLUG, and SNAIL [15,16,17], are activated to promote conversion of epithelial cells to a partial EMT state. Partial EMT cells can be viewed as a hybrid of epithelial and mesenchymal cells. They also have loose cell-to-cell connection, which allow them to migrate to wounding site. This process is reversible, so the partial EMT cells turn back to the epithelial phenotype after the injury site has been healed and the EMT triggering signal has been withdrawn [17].

Studies also support that EMT and MET take place during cancer metastasis. Some hypotheses regard cancer as an overhealing wound [18] or an abnormal development process [19], while there exist features specific for cancer progression. Invasion and metastasis are decisive steps in cancer progression and the major cause for cancer-related mortality [20]. At the cellular level, EMT or partial EMT leaves cells loosely connected to others, and enables them to depart from the primary location and migrate along the circulatory system to a secondary location, where the migratory cells can go through MET to epithelial cells again, proliferate, and form a secondary tumor [21]. Recent studies suggest that partial EMT rather than full EMT may play key roles on cancer metastasis [22,23,24].

Though EMT in breast cancer was first observed in 1890s [25], it did not attract attention in the carcinoma biology community for almost the whole past century. Only during the past decades researchers discovered many crucial signaling pathways and core regulatory elements that induce or contribute to EMT [8,26]. Like in embryonic development, metastasis in cancer progression can also be induced by various signaling molecules or cytokines [27], such as proteins in the TGF-β superfamily [28], hedgehog (HH) family, WNT family [29], and interleukin (IL) family [30], *etc*. Stressful microenvironments, such as hypoxia [31] or free radicals [32], also trigger EMT [33].

## 2. Multiple Signal Transduction Pathway and Their Crosstalks

**Figure 1:**
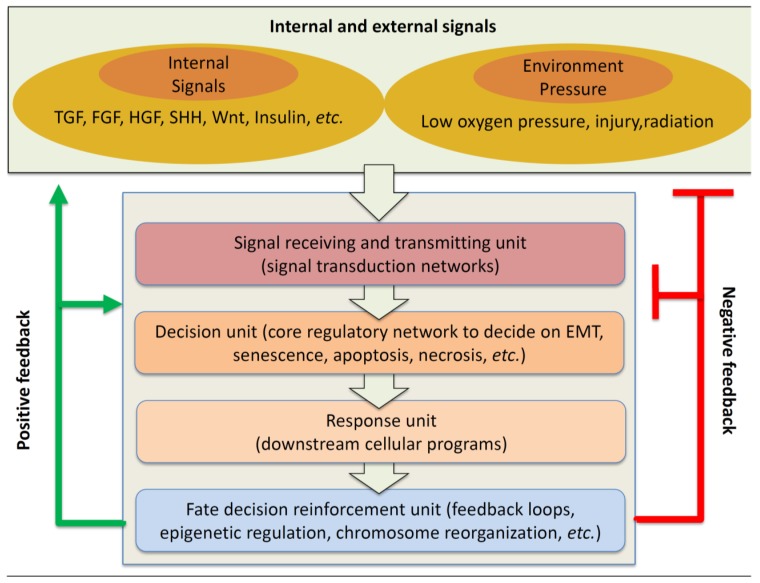
Schematic of the overall signal reception, transduction, and response process of EMT. Intracellular and extracellular signals are relayed via four basic units and regulated by positive and negative feedback loops.

Figure 1 schematically summarizes the overall cellular process of sensing, relaying, and responding to the stimulating signals. A plethora of stimuli activate multiple signal transduction pathways, which then converge to a core regulatory network composed by transcription factors (such as SNAIL1/2, ZEB1/2, TWIST) and miRNAs (such as miR34 and miR200 families) [34]. The latter further interact with other regulatory elements to instruct a cell to choose one of the several possible cell fates. For example, SNAIL1 can bind to P53, a major regulatory protein that induces senescence or apoptosis, and trap free P53 in the cytosol. Thus, SNAIL1 inhibits the choice of senescence or apoptosis [35]. Subsequently the fate decision is carried out by activating the corresponding expression program, and is strengthened by a series of events such as epigenetic modifications and activation of a number of feedback loops. The above flow-of-information is not unidirectional, but at every stage, there exists negative and positive feedbacks to previous stages, and form a closed network.

The TGF-β, Sonic Hedgehog (SHH), and WNT pathways are three well-studied signal transduction pathways that can induce EMT. Below we will give a general review on how these three pathways participate in signal sensing and processing during EMT, and how the knowledge has been applied to carcinoma treatment. Especially we hope to provide a perspective on the signaling network based on systems biology approaches, and insights on biomedical interventions of EMT.

### 2.1. TGF-β Pathway

TGF-β is a type of secretive protein, and it affects the cell that secrets the protein (autocrine) as well as its neighboring cells (paracrine). In human cells, three TGF-β isoforms (TGF-β1, TGF-β2 and TGF-β3) have been discovered, which share over 70% homological sequence. There are two types of transmembrane TGF-β receptors (TGFBR). Generally, first the type II TGFBR (TGFBR-II) molecules recognize and bind to TGF-β. Next, the complex recruits other TGFBRs and forms a complex with a stoichiometry of two copies of TGFBR-I, two copies of TGFBR-II, and one copy of active TGF-β. Formation of the complex activates the phosphorylation function of the intracellular part of TGFBRs to relay the TGF-β signals downstream of the pathway. The canonical pathway of TGF-β involves phosphorylation and activation of SMADs. Meanwhile, TGFBRs can also phosphorylate other signaling proteins, such as kinases, or transmit the signals via crosstalking to other pathways [36].

Paradoxically, TGF-β functions as both carcinomatous repressor and activator. More specifically, in normal cells or even some pre-cancer cells, TGF-β promotes proliferation arrest and, thus, represses tumor growth. However, in advanced malignant carcinoma cells, TGF-β promotes EMT and tumor metastasis. These seemingly contradictory functions come from the sophisticated regulation network among two types of parallel TGF-β pathways [37,38].

#### 2.1.1. SMAD-Dependent TGF-β Pathway

**Figure 2:**
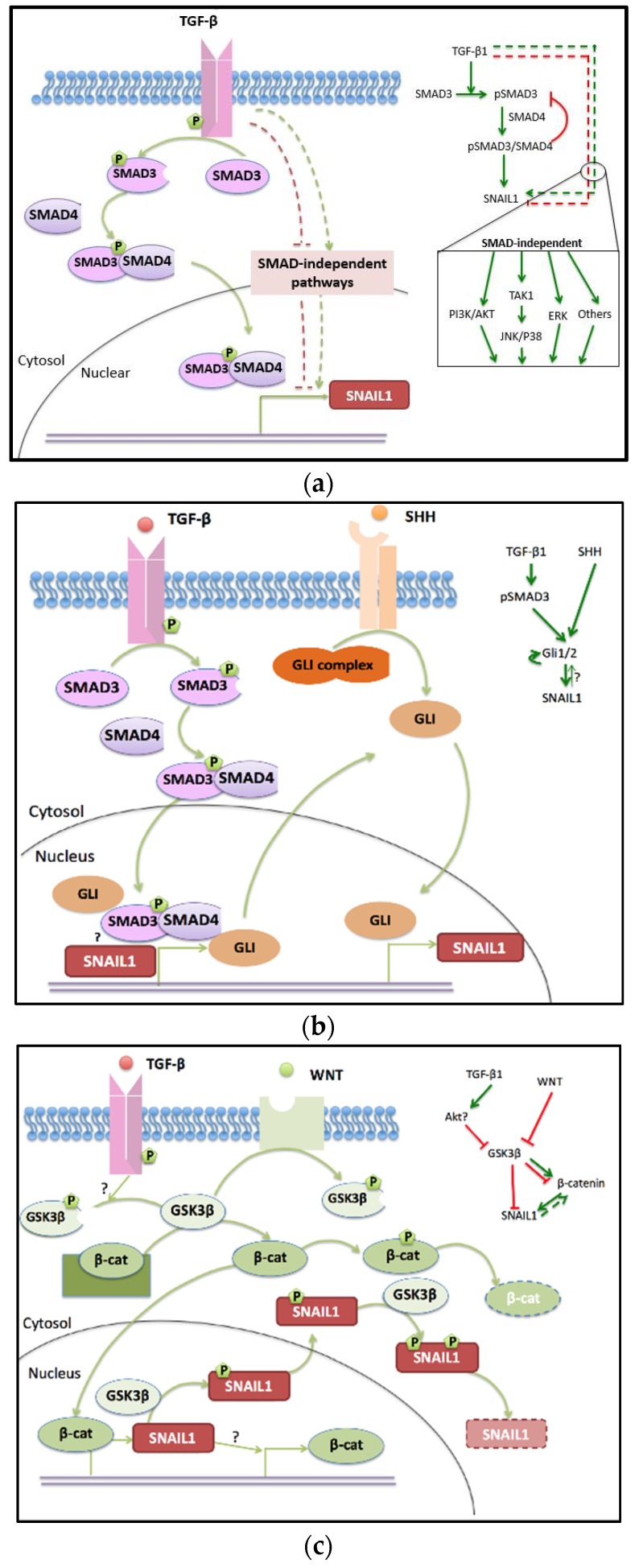
Crosstalk among the TGF-β (**a**, proteins in pink), SHH (**b**, proteins in orange), and WNT (**c**, proteins in green) signaling pathways converging to the core regulation unit. In the inserted regulation networks, point arrows represent activation, blunt ones represent inhibition, and dashed lines represent indirect links.

The more canonical TGF-β pathway depends on activation and deactivation of a SMAD family (Figure 2a). In this pathway, signaling is cooperatively regulated by three types of SMAD proteins in this family. Receptor-regulated SMADs (R-SMADs) include SMAD1, SMAD2, SMAD3, SMAD5, and SMAD8. They receive signals transmitted from the membrane-embedded signal receptors, and get phosphorylated [39]. Then, two molecules of phosphorylated R-SMADs come together and recruit a common-mediator SMAD (co-SMAD), SMAD4, to form a trimer, which can be transported from the cytosol into the nucleus [40,41,42]. In the nucleus, the heterotrimeric complex binds to DNA-sequence-specific transcription factors, individually or with other co-activators, to activate transcription of target genes [43], such as snail1 [44,45], snail2 [46,47], and other oncogenes. Inhibitory SMADs (I-SMADs) have two members, SMAD6 and SMAD7. They are downstream targets of TGF-β signaling, then negatively regulate activities of R-SMADs and co-SMADs [48], thus compose a negative feedback loop within the SMAD-dependent TGF-β pathway [49,50]. For instance, the active form of SMAD6 competes with SMAD4 on target gene binding and thus prevents the targeted genes of SMAD4 from being transcribed [51]. Similarly, the active form of SMAD7 interferes with activation of TGF-β receptors and phosphorylation of R-SMADs [52].

Elevated TGF-β-induced SMAD activation has been widely considered as a tumor promotion event, especially in highly malignant cancer cells. On the other hand, many reports documented that TGF-β-induced activation of SMADs also suppresses tumor formation and development by blocking the cell cycle and arresting cell growth [53] in breast cancer cell lines [54], liver cancer cells [55], and normal epithelial cells [56]. Mutations on smad2 and smad4 have been reported in colorectal cancer [57], pancreatic ductal adenocarcinoma [58], or hepatocellular cancer [59]. These mutations implicate the potential antitumor function of SMADs [60,61]. Moreover, depending on cell types, TGF-β induced SMAD signals can also induce apoptosis as a safeguard mechanism to prevent transformed cell from EMT or metastasis [62,63].

The diverse roles of SMAD activation come from how the SMAD2/3/4 heterotrimeric complex performs its function. When the SMAD trimer acts alone, some of the targeted genes can be activated only at a low basal level. More efficient activation of these genes requires binding of the trimer to other sequencing-specific activators [64]. For example, the SMAD2/3/4 complex recruits CBP/P300 as the co-activator to activate p15 [65] and p21 [66], both of which inhibit cells from progression of cell cycles [67]. On the other hand, if the heterotrimer recruits EMT promoting transcription factors as the co-activator, such as TWISTs, it can up-regulate oncogene expression [68]. This cofactor-dependent gene expression pattern explains why TGF-β functions differently in cells of different type and cell stage, and the outcome is affected by the existence of other stimuli.

#### 2.1.2. SMAD Independent Pathway

Other than the SMAD-dependent pathway, TGF-β receptors also relay the signals through a group of additional signal-transmitting proteins, such as PI3K/AKT, MEK/ERK1/2 [69], RHO-A, and JNK/P38 [70]. Compared to the SMAD-dependent pathway, the SMAD-independent pathways are even more complicated, with crosstalks among the signaling proteins forming an intricate molecule-molecule interaction network. For instance, TGF-β can induce AKT phosphorylation and activate phosphatidylinositol 3-kinase (PI3K) rapidly, which possibly contributes to SMAD2-induced EMT [71]. Studies on keratinocyte cells show that the PI3K/AKT pathway helps to complete the TGF-β-induced SMAD-dependent EMT [72]. Furthermore, PI3K and AKT antagonize TGF-β-induced apoptosis and growth arrest [73,74], and bias TGF-β-treated cells to undergo EMT. Similarly, the TGF-β-induced ERK/MAPK pathway contributes to EMT induction, since ERK is required for removing cell adherens junctions to increase cell mobility. TGF-β also activates RHO-like GTPases in the RHO pathway, which have multiple functions on cytoskeletal organization, apoptosis, and EMT [75], as well as the RHO-A-dependent signaling pathway to promote mesenchymal characteristics in epithelial cells through inducing stress fiber formation [70,76]. Another TGF-β induced SMAD-independent pathway is the JNK/P38 pathway via the activation of TGF-β activated kinase 1 (TAK1). JNK itself can phosphorylate R-SMAD directly, thus, turning on the EMT program. Both JNK and P38 can work synergistically with SMADs to promote TGF-β induced apoptosis [70,77].

Put all of the above together, at pro-oncogenic stage, TGF-β has opposing effects on tumor development. Some of the SMAD-independent pathways have tumor-suppressing functions, such as promoting apoptosis. However, the SMAD-independent pathways can also promote tumor development through suppressing apoptosis and promoting EMT [70]. Physiologically, at the pre-tumor stage the SMAD-dependent pathway promotes cell cycle arrest and apoptosis to stop tumor formation and growth. In malignant cells, when the amount of cofactors of oncogenes exceeds that for apoptosis or growth arrest, TGF-β functions more as an EMT-inducer through both SMAD-dependent and SMAD-independent pathways. Therefore, in cancer progression, the relative balance between SMAD-dependent and -independent signal transduction likely plays a critical role on determining cell fates [70].

#### 2.1.3. Drugs Targeted to TGF-β Pathway

Based on its cancer-promotion function, members of the TGF-β family are potential drug targets for clinic therapy. Moreover, given the fact that the TGF-β pathway involves in DNA damage repair [78], inhibition of TGF-β signaling may enhance the efficacy of radiotherapy and chemotherapy [79]. Popular clinical treatments that target to blocking TGF-β signaling include trapping TGF-β ligands, blocking the receptor kinases signaling, using antisense oligunucleotides to decrease the translation of TGF-β protein, and using peptide aptamers to block the transduction pathway [80]. For example, to trap TGF-β molecules from binding to TGFBRs, 1D11 [81], and GC-1008 (Fresolimumab) [82] have been generated as TGF-β neutralization monoclonal antibodies and used in treatment of melanoma [83] and glioblastoma [84]. AP12009 (Trabedersen) is designed to inhibit TGF-β2 expression and has been used to treat pancreatic cancer [85]. Small chemical molecules are developed to block TGF-β signaling by inhibiting the phosphorylation function of TGFBRs, among them SB431542 is widely used to inhibit TGFBR-I in breast cancer therapy [86,87] and LY2109761 is another TGFBRs inhibitor that is applied in treating pancreatic cancer [88]. Antibodies raised against targeted proteins in TGF-β pathways can also be used as therapeutic approach. For example, the primary tumor growth are proven to be arrested by TGFBR-II antibodies [89]. Development of peptide aptamer drugs is a new direction that is still at its early stage. Some peptide aptamers targeting R-SMADs or co-SMAD have been discovered and tested in cell lines [90]. Since they target specific proteins, using peptide aptamers might be a more promising clinical therapy strategy considering the dual roles of TGF-β. By blocking only one sub-TGF-β-pathway, one can possibly suppress the tumor-promotion function of TGF-β without removing its anti-tumor benefits.

Therapeutic blockage of TGF-β signaling is tricky due to the pleiotropic effects of TGF-β on tumor progress. As a secretive protein, controlling the TGF-β signaling microenvironment near carcinoma is as important as controlling the intracellular TGF-β signaling pathway. Moreover, TGF-β has important regulatory functions on normal cell physiology. Complete blockage of the TGF-β pathway is detrimental to normal cells and thus not recommended. Therefore, while significant progress has been made on developing drugs that target the TGF-β signaling pathways, clinically, these drugs should be used with caution and perhaps only in certain cancer types.

### 2.2. SHH Pathway Engages in EMT and Crosstalks to TGF-β

In addition to the TGF-β pathway, the SHH pathway has been reported to induce EMT individually, or cooperated with other pathways in lymphatic and gastric tumors [91], pancreatic cancer [92], breast cancer [93], *etc.* (Figure 2b).

Like the TGF-β pathway, the SHH pathway starts from secretive hedgehog proteins, a family of glycoproteins. The precursors of SHH proteins undergo several steps of post-translational modification and cleavage before maturation, which are then secreted as oligomers or soluble multimers, and can diffuse over various distances between tissues in the body before being removed [94]. SHH belongs to the hedgehog (HH) family. Three mammalian HH proteins have been identified in the HH family recently, sonic hedgehog (SHH), Indian hedgehog (IHH) and desert hedgehog (DHH). While these three HH proteins share some redundant functions, each of them also has evolutionarily-specified roles. For example, Sonic Hedgehog (SHH), the most common and understood one, is crucial in embryo development, cancer progression, and body patterning [95,96].

#### 2.2.1. The off and on States of the SHH Pathway

The canonical SHH pathway can be generally divided into three parts; the signal reception elements, the signal transmission elements, and downstream transcription factors. In the absence of SHH, the pathway is at an “off” state. HH-patched protein (PTC), which is the transmembrane receptor element, binds to smoothened protein (SMO) to inhibit SMO activation. In the cytosol, activated protein kinase A (PKA) binds to other kinases including glycogen synthase kinase 3β (GSK3β) and other factors to phosphorate glioma-associated oncogene homologs (GLIs). In the absence of SHH signals, GLIs have only low basal level expression, and the proteins assume a repressor form. That is, GLIs repress the expression of their target genes. When SHH is present, the pathway switches to an “on” state. SMO proteins are released and phosphorylated to promote the activation of GLIs [97,98,99]. Some GLI proteins (e.g., GLI2) are truncated at the carboxy-terminal by the proteasome and turn to the activator form [100,101], which then activate the expression of their target genes.

#### 2.2.2. Regulation of GLI Proteins and Crosstalk to the TGF-β Pathway

GLI proteins are the major transcription factors in the SHH pathway. A high level of GLI proteins indicates activation of the SHH pathway [102]. Three types of GLI proteins have been identified in mammals, GLI1, GLI2, and GLI3. Though they share long homologue sequences, and also have similar DNA-binding sequences, they play quite different roles in development, EMT, and cancer promotion. In addition to the DNA binding domain, GLI1 has only the activator domain and can be activated by SHH. GLI2, and GLI3 have both the repressor domain and the activator domain. However, in most contexts, SHH activates GLI2 [103], while it is unclear SHH activates or represses GLI3 [104].

Activated GLI1 and GLI2 can directly promote the expression of a group of genes by physically binding to their promoter region, including oncogenes and genes that are involved in the EMT process [105], such as bmi1 [106], nanog [107], snail1 [108,109]. Based on the fact that expression of GLI1 can be regulated by the E-box [110], positive feedback loops may exist between GLI1 and its target transcription factors that contain E-box at the promoter region of their genes, such as SNAIL1. Furthermore, GLI proteins can also be up-regulated by SMAD proteins [111,112]. Actually, the TGF-β/SMAD/GLI2 axis has been suggested to be essential for cancer metastasis [113]. Consequently, the SHH pathway and the TGF-β pathway crosstalk to each other and coordinately induce EMT. GLI proteins are also involved in several positive or negative feedback loops within the SHH signaling pathway. For example, the activated form of GLI2 can directly bind to the promoter region of gli1 to up-regulate GLI1 protein expression, while GLI1 can also induce GLI2 expression directly or indirectly, so the two form a positive feedback loop [103,114]. On the other hand, GLI1 induces PTC, and PTC inhibits GLIs to form a negative feedback loop [103].

#### 2.2.3. Clinical Observation and Interventions of SHH Signaling Pathway in Cancer

There are clinical reports on abnormal activation of the SHH signaling pathway in different types of cancer. For example, in thyroid cancer, SHH is expressed in 64% of PTC tissues but only in 17% of non-cancerous tissues, and GLI1 is expressed in 48% and 9% of these two different types of tissues, respectively [115]. Activation of the SHH pathway is related to promotion of the EMT process in lung cancer cell lines [116], renal cell cancer [117], and gastric cancer [91]. Based on these observations, blocking SHH signaling is a popular strategy in cancer therapy. Indeed, inhibition of SHH signaling can reduce the proliferation rate of non-small-cell-lung-cancer cells significantly [118]. A similar phenomenon has also been observed in breast cancer [119].

A basic strategy of intervening the SHH pathway is blocking the SHH receptor or other major players downstream in this pathway [104]. In pancreatic cancer therapy, combination of a SMO inhibitor, cyclopamine, and gemcitabine, a nucleotide analog, completely abrogate pancreatic cancer metastasis while also significantly reduce the size of the primary tumor [120]. Cyclopamine, vismodegib, and other SMO inhibitors have been used widely in clinic for medulloblastoma [121], ovarian cancer [122], and pancreatic cancer [123] treatment. Given the importance of GLI1/2 in the SHH pathway on promoting EMT and metastasis, blockade of GLI1/2 is a candidate for cancer treatment. For example, small chemical molecules, GANT58 and/or GANT61, which block GLI1/2 function, arrest prostate tumor growth [124]. Compared to blocking the upstream regulators in the SHH pathway, an advantage of targeting GLI proteins is that these proteins serve as signaling hubs of multiple pathways that are activated in cancer cells, such as the TGF-β, WNT, and SHH pathways.

### 2.3. WNT Pathway in Cancer Progress and EMT

The WNT pathway is another signaling pathway that crosstalks to the TGF-β pathway and promotes EMT. The tumor repressor GSK3β, and the activator β-CATENIN are two major converging elements between the WNT and TGF-β pathways (Figure 2c).

The WNTs comprise a large family of proteins that are highly conserved from fruit fly to human [125]. In *homo* species, 19 discovered WNTs compose a very intricate network, which is essential for development and stress responses. Abnormal activation or mutations in the WNT pathway has been reported in many cancer types, such as intestinal neoplasms, breast cancer, prostate cancer, and lung cancer [126].

The canonical WNT pathway starts from reception of signaling molecules on the cell membrane. GSK3β is a major downstream regulator of the receptors. Without WNT signals, GSK3β keeps its active form, which can phosphorylate its target proteins (e.g., β-CATENIN [127]) for further degradation. When the WNT signaling pathway is activated, GSK3β is phosphorylated to an inactive form. Thus, functional β-CATENIN is accumulated in the cytosol and is further transported into the nucleus. In the nucleus, together with TCF/LEF, β-CATENIN binds to the promoter region of a target gene, such as SNAIL1, and activates its transcription [9,128]. Furthermore, SNAIL1 can also form a positive feedback with β-CATENIN by interacting with the β-CATENIN physically [129], or increase the amount of free β-CATENIN indirectly through EMT process [130]. Inactivation of GSK3β can also increase SNAIL1 expression directly following two steps: in the nucleus, it is phosphorylated by GSK3β; then SNAIL1 can be transported from the nucleus to the cytosol, where it can be phosphorylated again by GSK3β for final degradation [131].

The WNT pathway affects and is affected by several signaling pathways, including the SHH and TGF-β pathways. GSK3β affects GLI proteins both positively and negatively. On one hand, GSK3β phosphorylates GLI proteins for degradation [132]. On the other hand, GSK3β phosphorylates SUFU, a scarf protein for GLI proteins, and releases free GLI proteins [133]. GSK3β also stabilizes GLI mRNA indirectly, leading to an increase of the amount of GLI proteins [134]. Subject to TGF-β stimulation, the WNT pathway can be activated by SMAD-independent pathways. For instance, in human lung fibroblast cells, TGF-β1 can inactivate GSK3β by activating the mitogen-activated protein kinase (MAPK) pathway and phosphorylating ERKs [135]. GSK3β can be inhibited by the ARK pathway, and the latter can be activated by TGF-β1 in some cell lines. In addition, GSK3β negatively affects the TGF-β pathway by phosphorylating SMAD3 with its cooperator, AXIN, and triggers its ubiquitination and degradation when TGF-β is absent [136].

#### Clinical Observation and Interventions of the WNT Signaling Pathway in Cancer

Based on the well-documented close relationship between WNT signaling aberrance and cancer, intensive efforts have been put on designing drugs that specifically target the WNT pathway [137]. However, no drug has been approved for clinical usage yet [138]. Special caution has to be taken since the WNT pathway has important functions in almost every aspect of mammalian cells, such as proliferation and regeneration.

In addition to target-specific small molecules, a group of drugs and compounds widely used for other purposes have been proven to help in cancer treatment as they also block the WNT pathway. For example, aspirin affects and blocks the WNT pathway as a non-steroidal anti-inflammatory drug (NSAID) at multiple levels, such as facilitating β-CATENIN degradation [139]. Vitamins, such as retinoids, vitamin D, *etc.*, show effects in colorectal cancer and breast cancer probably through interacting with β-CATENIN and TCFs [137].

## 3. Systems Biology in Signaling Crosstalk and Drug Discovery

As we discussed above, the crosstalk network among the TGF-β, SHH, and WNT signaling pathways is complex. In addition, some of the signals, such as TGF-β, have opposite roles as both cancer repressor and promoter, depending on cell types and cancer stages. Similarly, some of the regulators can both turn “off” and “on” their target genes. For example, GLI proteins can both negatively and positively regulate expression of themselves. Another typical example is GSK3β, which can covalently modify both oncogene proteins (e.g., SNAIL1) and tumor repressors (SUFU) for degradation [133]. Furthermore, all of these signaling pathways have essential roles on both normal cell life cycle and in cancer development.

Due to the above-mentioned molecular biology complexity, a naive drug-design strategy based on simply blocking certain pathway likely has serious side effect to normal cells and patients. Cancer therapy and anti-tumor drug discovery are, thus, difficult, time consuming, and face three basic challenges:

1. Which of the proteins/regulators to target for clinic and commercial consideration?
2. How to select chemicals that are suitable for therapy from the gigantic data pool?
3. How to design treatments that target to specific population of tumors in patients?

Currently computational and system biology studies become indispensable on revealing the molecular mechanism and addressing the three challenges. These studies engage widely in current molecular biology, and provide systematic and integrative perspectives on understanding the biological implications underlying individual experimental results. In the next section we will use a series of recent studies to illustrate how we can advance our understanding of the EMT regulatory mechanism through combined mathematical modeling and experimental studies.

**Figure 3:**
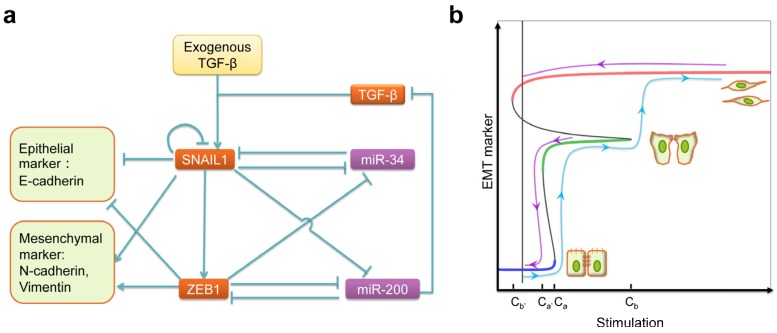
Systems biology study on the core EMT network. (**a**) Core regulatory network of TGF-β induced EMT revealed by experimental studies. Point arrows represent activation, and blunt ones represent inhibition; and (**b**) mathematically-predicted bifurcation diagram. Also shown are the corresponding dose-response (D-R) curves that are more familiar to experimentalists. Notice that the D-R curves are different for cells starting from different phenotypes and treated with increasing (blue curve) and decreasing (purple curves) exogenous TGF-β, respectively. This history-dependent hysteresis is a signature of bistable dynamics. The predicted bifurcation diagram has been experimentally confirmed in MCF10A cells. Adapted from [142,144].

With increasing reports on new signals and pathways leading to EMT, one might have the impression that EMT can be induced easily. Actually, EMT is tightly regulated at multiple levels, and pathological EMT is a rare event in a healthy body. Furthermore, EMT is not a “to be or not to be” question. Instead, EMT proceeds through a wide spectrum of intermediate states, generally referred as the partial EMT state [140,141]. Tian *et al.* [142] mathematically analyzed the core EMT regulatory network (Figure 3a), and proposed a sequential two-step mechanism, as summarized in Figure 3b. A SNAIL1/miR34 double negative feedback loop and a ZEB1/miR200 feedback loop form two binary switches. In epithelial cells, both miR34 and miR200 are highly expressed, while SNAIL1 and ZEB1 express only at basal levels. With an intermediate concentration of TGF-β (exceeding a threshold value C_a_), snail1 transcription is activated. The protein product SNAIL1 further inhibits transcription of miR34 [143], partially up-regulates epithelial markers such as E-cadherin and down-regulate mesenchymal makers such as Vimenten and N-cadherin. At this stage, the ZEB1/miR200 switch has not been reverted, and cells exist in a partial EMT state. Experimentally, if one now reduces the exogenous TGF-β to a lower level (below a threshold value C_a’_ < C_a_), cells returns to the epithelial state. That is, the Epithelial-to-partial EMT transition is reversible under TGF-β treatment. When the exogenous TGF-β level exceeds a second threshold (C_b_ > C_a_), miR200 is degraded and the level of ZEB1/2 increases. ZEB further inhibits transcription of miR200 [141], works together with SNAIL1 and other factors to up-regulate epithelial markers and down-regulate mesenchymal makers, so cells undergo a full EMT. At this stage cells express autocrine TGF-β, which maintains cells in the mesenchymal state even when the exogenous TGF-β is removed (the crucial point C_b’_ < 0). That is, the full EMT is irreversible under TGF-β treatment. Subsequently, Zhang *et al.* [144] performed quantitative experimental studies using the human mammary MCF10A cell line, and confirmed all model predictions. Therefore, the studies reveal how different cell phenotypes emerge out of the interactions among the transcription factors and microRNAs, and provide clues for biomedical intervention to regulate the conversion between them.

The above-mentioned model clearly does not provide a complete picture on EMT but rather should be considered as a starting point. It only considers a small core network without explicitly considering many other key EMT players such as TWIST. Actually there are many more positive feedback loops formed by various regulating elements. These feedback loops can also form multiple stable switches [145], which may function either in synergy or in sequence to give rise to possibly a combinatorial number of partial EMT states, consistent with the notion of a quasi-continuum EMT spectrum [146]. Further experimental studies can also analyze whether the revealed two-step mechanism is general for different cell types and cell lines, and if not (which is very likely), what are the differences and common themes. While there are many possible directions for expanding the modeling efforts, below we discuss three of them.

First, the model can be systematically expanded to include other involved molecular species. Using a more coarse-grained Boolean network modeling framework, recently Steinway *et al.* studied a more complex network of EMT including crosstalks among the TGF-β, SHH, and WNT pathways, and tested model predictions experimentally [147,148]. Expansion of an ordinary differential equation based model like ours can more faithfully describe the temporal and steady state dynamics of the system. For this purpose systematic and quantitative measurements are needed to provide input for constraining model parameters and testing model predictions.

Second, the field awaits further methodology developments on incorporating high-throughput data into detailed dynamics modeling. The past decade has observed an explosion of accumulation of “omics” datasets, such as transcriptomics, epigenomics, proteomics, and metabolomics. Typically a high-throughput dataset provides a global view of a system or process under study, although at relatively low resolution, both in temporal features and in data quality, compared to a more focused study like the ones discussed above. Bioinformatics tools have been widely used to analyze the omics data. For example, Nam, *et al.* used PATHOME, an algorithm based on entire pathway information, to connect the WNT and AMPK pathways at HNF4a-WNT5A in gastric carcinogenesis, and further predict WNT5A as a suitable therapy target [149]. Given the importance of including dynamics into pharmaceutical development [150,151], the challenge is how to combine these global and focused levels of studies.

Third, we may observe more examples of integrating computational and systems biology approaches in new drug discovery, especially in screening the druggable structure of targeted proteins and selecting drug candidates [152]. Computational structure biology has already been used widely in drug candidate screening. For example, Baken *et al.* analyzed a pharmacophore model (PM), and selected seven compounds for further experimental screening out of over two million candidates [153]. The procedure can be more efficient and effective by placing drug discovery in the context of network dynamics.

In summary, EMT is a complex process and many pathways crosstalk extensively to initialize and regulate EMT. Therefore, integrated computational and experimental approaches are necessary to tackle the molecular and cellular regulation mechanisms, and optimize biomedical intervention strategies.
